# Cultivation of Liquorice in This Country

**Published:** 1856-04

**Authors:** 


*Cultivation of Liquorice in this Country.*—A correspondent of the New York Times (Mr. William R. Prince, of Flushing, L. I.) is of the opinion that the officinal Glycyrrhiza glabra may be easily cultivated in this country. He says: "The liquorice is one of the most important plants that is destined to be added to American agriculture, and merits at our hands an early adoption, on account of the facility of its culture, its great usefulness for various purposes, and for the large profit it yields to the cultivator. When the high-priced lands of England are profitably devoted to it, how much more profitable must it prove, where land is plentiful and cheap, and where, above all, as in several of the Western States, the soil is naturally permeable, free from all stones, and no manuring required. It is, indeed, mortifying to American pride, to witness the many thousands now paid to Europe for an article like this, so simple in its culture that we ought to be the largest exporters of it, thus adding another item to our 'granary of the world.'

"It has been extensively cultivated in Spain, and from the commencement of Queen Elizabeth's reign it has been largely grown in various parts of England."

Besides its employment in medicine, liquorice is extensively used in the manufacture of porter and other preparations containing saccharine ingredients, and its introduction into this country could not fail to be profitable.—*Boston Med. and Surg. Journal.*

